# Transmission of mycoviruses: new possibilities

**DOI:** 10.3389/fmicb.2024.1432840

**Published:** 2024-06-27

**Authors:** Živilė Buivydaitė, Anne Winding, Rumakanta Sapkota

**Affiliations:** Department of Environmental Science, Aarhus University, Roskilde, Denmark

**Keywords:** virus-host interactions, cross-kingdom interactions, vertical transmission, horizontal transmission, fungi

## Abstract

Mycoviruses are viruses that infect fungi. In recent years, an increasing number of mycoviruses have been reported in a wide array of fungi. With the growing interest of scientists and society in reducing the use of agrochemicals, the debate about mycoviruses as an effective next-generation biocontrol has regained momentum. Mycoviruses can have profound effects on the host phenotype, although most viruses have neutral or no effect. We speculate that understanding multiple transmission modes of mycoviruses is central to unraveling the viral ecology and their function in regulating fungal populations. Unlike plant virus transmission via vegetative plant parts, seeds, pollen, or vectors, a widely held view is that mycoviruses are transmitted via vertical routes and only under special circumstances horizontally via hyphal contact depending on the vegetative compatibility groups (i.e., the ability of different fungal strains to undergo hyphal fusion). However, this view has been challenged over the past decades, as new possible transmission routes of mycoviruses are beginning to unravel. In this perspective, we discuss emerging studies with evidence suggesting that such novel routes of mycovirus transmission exist and are pertinent to understanding the full picture of mycovirus ecology and evolution.

## 1 Introduction

Mycoviruses are mostly RNA viruses and have been reported as asymptomatic to the host, hardly causing any effect on host phenotype (Ghabrial et al., 2015). While the mycoviruses are widespread in all major clades of fungi (Gilbert et al., 2019), it is only in recent years with the advent of high throughput sequencing and the rapid development of bioinformatics tools, that ecological studies of mycoviruses and their diversity have become possible (Babaian and Edgar, 2022). Despite that most mycoviruses have a neutral or no effect on the host, a few viruses have been shown to negatively affect fungal growth or virulence, a phenomenon termed hypovirulence (Nuss, 2005). A well-studied example of hypovirulence involves the pathogenic fungus *Cryphonectria parasitica* and its mycovirus, Cryphonectria hypovirus 1 (CHV1), which significantly reduces the pathogenicity of the fungus in chestnut trees (Dawe and Nuss, 2001). This case is often portrayed as a success story of biological control by mycoviruses (Milgroom and Cortesi, 2004). In recent years, there has been a growing number of reports on fungal hypovirulence, emphasizing the significant potential of mycoviruses as biocontrol agents against pathogenic fungi (García-Pedrajas et al., 2019).

The complex dynamics of mycovirus-fungus interactions can lead to a multitude of potential effects, which increasingly captured the interest of the scientific community (Villan Larios et al., 2023). In nature, fungi are frequently exposed to several mycoviruses at once, and several studies focusing on plant pathogens have reported a number of mycoviruses, suggesting their potential significance in crop health (García-Pedrajas et al., 2019). However, the detection of only a limited number of mycoviruses exhibiting hypovirulence may be attributed to the culturing of fungal isolates in the laboratory. Unknowingly, the natural viral load of the fungus might either be lost or limited to only beneficial or weak viruses by our screening for healthy individual fungal cultures and subculturing the fungi through several generations of growth in the laboratory. Further, while the fungal kingdom is diverse, a large number of mycoviral studies are focused on a few fungi with economic importance such as plant pathogens (Wagemans et al., 2022). Compared to viruses infecting other groups such as plants and animals, mycoviruses remain understudied, especially in terms of their transmission routes (Ghabrial et al., 2015).

Transmission of mycoviruses is of great importance and significance to understand their ecology and role in regulating fungal populations, and efficient mycovirus transmission is a core requirement of a successful biological control agent (van Diepeningen, 2021). However, the mechanisms by which mycoviruses transmit have only begun to be unraveled over the past decade. The traditional view of mycovirus transmission primarily emphasizes vertical routes via spores and horizontal transmission through hyphal contact. This perspective has been challenged in recent years. This notion is based on the fact that mycoviruses have no extracellular route of transmission, with the exception of a single case involving DNA mycovirus Sclerotinia sclerotiorum hypovirulence-associated DNA virus 1 (SsHADV1) (Yu et al., 2013). SsHADV1 is not only infectious when applied extracellularly, but it is also capable of reducing virulence in several host fungi (Yu et al., 2013). It remains unclear if extracellular infection of DNA mycoviruses occurs under natural conditions. Further research is needed to understand the prevalence of this phenomenon and whether other DNA mycoviruses can also infect extracellularly. Nevertheless, the capacity for extracellular infection is a major advantage for a potential biocontrol agent, allowing for simpler application and faster spread.

The widespread occurrence of capsidless RNA viruses, including several members of mycoviruses, further indicates that RNA mycoviruses rely exclusively on intracellular routes for transmission. However, currently known transmission routes of vertical and horizontal transmission only partially account for the existing high diversity of mycoviruses. Reliance on these mechanisms alone implies that only closely related fungi can exchange viruses, leaving aspects of mycovirus diversity unexplained. Much like plants and animals, fungi are exposed to diverse mycoviruses, suggesting that movement and transmission may occur via other routes. Hence, researchers are pointing out the possibility of other mycoviral transmission pathways (Myers and James, 2022) based on analyses of multiple mycovirus genomes indicating retention of the genes coding for the coat protein, and the presence of mycovirus particles which has been detected in numerous instances (Dolja and Koonin, 2018; Hough et al., 2023). New potential transmission pathways could explain the diversity of mycoviruses and the dilemma that vertical transmission will only support mycoviruses causing positive effects, while harmful mycoviruses would lead to a reduction in host fitness, and eventually death of both the fungus and the mycovirus. However, in nature, we find beneficial, neutral, as well as harmful mycoviruses.

Recent reports indeed reveal new transmission routes, indicating a broader range of horizontal transmission mechanisms not limited to hyphal anastomosis. For instance, studies have reported insect-mediated transmission of mycoviruses (Liu et al., 2016) and mycoviruses able to replicate in plant cells (Nerva et al., 2017), making them potential vectors, and therefore, suggesting alternative routes of transmission. Additionally, reports propose that viral particles can directly infect fungi (Yu et al., 2013). This could hold true not only for the exceptional DNA mycoviruses, but for the RNA viruses too, where virion particle infection was reported with success (Urayama et al., 2010). This indicates that extracellular, horizontal infection of mycoviruses might occur in nature with varying degrees of efficiency, but the duration of mycovirus infectivity in the extracellular phase remains unknown. Overall, these recent developments challenge our understanding of the origin of mycoviruses and point to a need for further investigation into their transmission routes and potential vectors. Finally, we have limited knowledge on how newly acquired mycoviruses alter or impact fungal host phenotype, as well as how these mycovirus-fungus interactions extend further, for example, to plant-fungus interactions.

**Figure 1:**
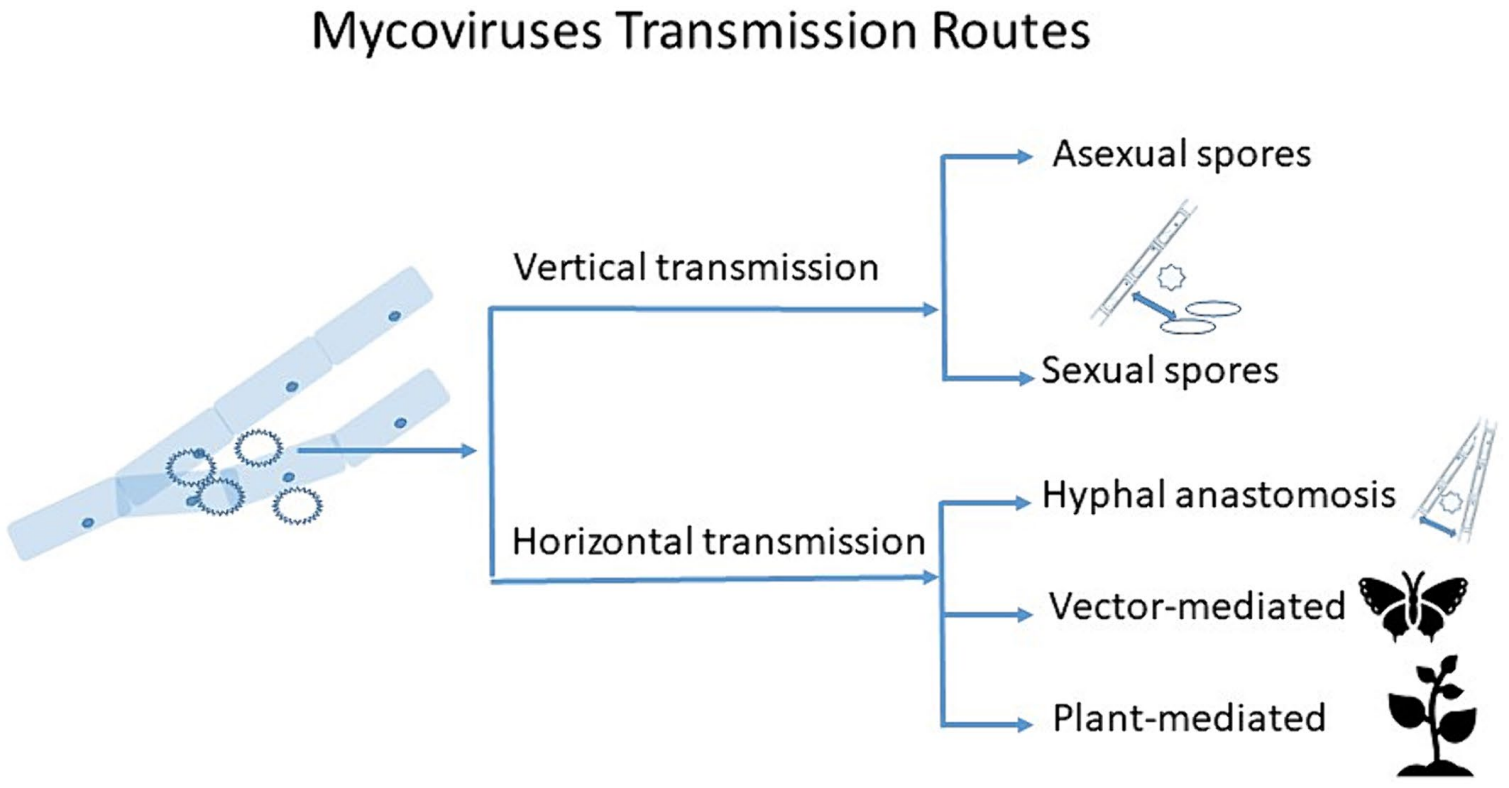
Schematic diagram showing transmission routes of mycoviruses. Vertical transmission occurs through sexual and asexual spores, with varying degrees of efficiency. Horizontal transmission occurs via hyphal anastomosis between vegetatively compatible isolates, but vector- and plant-mediated horizontal transmission might take place too, which can also be extracellular.

Here we outline major known transmission routes of mycoviruses, and discuss the potential new avenues for mycovirus transmission research (Figure 1).

## 2 Vertical and horizontal mycovirus transmission via hyphal contact: the established pathway

Fungi have evolved to reproduce via asexual and sexual spores, and mycovirus infection is often based on vertical transmission. In stable and resource-rich environments, fungi mostly rely on asexual reproduction, and in some fungi, asexual spores are more common than sexual spores, while for some, sexual reproduction has never been observed. Likewise, mycovirus transmission is thought to be more common through asexual spores than sexual, but the evidence is inconsistent. For example, the previously mentioned *C. parasitica* is known to produce both sexual and asexual spores, but CHV1 is exclusively transmitted via asexual spores, which is not the case for other mycoviruses like Mycoreovirus 1 (MyRV1) and MyRV2 (Deng et al., 2007). On the contrary, *Fusarium graminearum*, which can also produce both sexual and asexual spores, showed 100% efficiency to sexual spores of several dsRNAs (Chu et al., 2004). Therefore, it is evident that vertical transmission has mixed transmission success, with efficiency depending on the specific fungus-mycovirus combination (Ihrmark et al., 2002; Romo et al., 2007; Herrero and Zabalgogeazcoa, 2011). In principle, efficient vertical transmission alongside other routes, such as horizontal transmission, would naturally lead to the accumulation of mycoviruses in fungi. Remarkably, emerging data consistently supports the notion that multi-viral infections are more common than previously presumed (Osaki et al., 2016; Tran et al., 2019; Myers et al., 2020). However, mycoviruses might be lost during spore formations due to varying viral transmission efficiency and virus-free fungi might have phenotypic advantages, leading to a hypothesis that fungi may selectively lose harmful viruses while retaining neutral or beneficial ones.

Horizontal transmission enables the transfer of mycoviruses across vegetatively compatible strains via cell-to-cell contact in which hyphae connect, a mechanism referred to as hyphal anastomosis. This process allows for the transfer of cytoplasm, along with the viruses, from one fungus to another. Vegetative compatibility is commonly observed only between closely related strains, as it is a complex mechanism, governed by multiple vegetative incompatibility genes, which provide fungi with a self/non-self-recognition system. Horizontal transmission via anastomosis is now considered a well-documented route, and transfer of mycoviruses horizontally has been successfully reported not only in *C. parasitica*, but also in many other fungi (Papazova-Anakieva et al., 2008; Sun et al., 2023). Such transfer of mycoviruses may be only unidirectional and likely depend on the donor fungi (Carbone et al., 2004). One strategy some mycoviruses employ to facilitate transmission is to weaken the incompatibility reactions between the host and the recipient fungus (Biella et al., 2002; Wu et al., 2017), but more research is needed to assess the frequency of this strategy.

Interestingly, it is estimated that horizontal transmission of mycoviruses occurs at higher rates at natural field conditions than in laboratory settings (Brusini and Robin, 2013). This could be attributed to several factors, with a plausible explanation being that in natural environments, niche-based growth of certain fungal groups brings multiple fungal isolates into close proximity, along with other organisms, such as arthropods and plants. These interactions, or specific environmental conditions, could favor horizontal mycovirus transmission from one isolate to another or from one fungal species to another through yet unknown mechanisms. One contributing factor may be associated with zinc ions, an essential micronutrient in plants. Its addition to the growth medium has been shown to promote hyphal anastomosis and mycovirus transmission; however, the exact mechanism remains unclear (Ikeda et al., 2013).

## 3 Vector-mediated transmission of mycoviruses: uncharted territory

Vector-mediated viral transmission involves insects, arthropods, nematodes or other animals which act as intermediators in the transfer of viruses to another host. Vector-borne transmission in plant and animal viruses is broadly categorized as non-persistent and persistent based on the time period the viruses are transmitted after being acquired by the vector. In non-persistent vector-borne transmission of viruses, the viruses are limited to the mouth part of the vector enabling short-term transmission, and are not able to replicate in the vector. In contrast, in persistent vector transmission, also referred as circulative, viruses can cross the intestinal barrier of insects or arthropods, and can thus be retained in the vector for extended periods or even replicate within the vector, facilitating long-term transmission. We hypothesize that vector transmission of mycoviruses might have evolved in a similar mechanism given the diversity of fungivores in nature that feed on fungi. Although numerous fungivorous insects, mites, nematodes, protists, and other animals are well-known, researchers doubt if they act as mycoviral vectors. One argument against vector-mediated transmission is that mycoviruses generally lack vector transmission-associated proteins found in plant viruses (Bragard et al., 2013). For example, glycoproteins mediate virus entry into insect vectors in many negative-strand plant RNA viruses. However, glycoprotein genes are absent in negative-strand fungal RNA viruses, with notable exceptions such as Fusarium asiaticum mycobunyavirus 1 (Huang et al., 2023) and Sclerotinia sclerotiorum rhabdovirus 1 (Mu et al., 2021). Nevertheless, given that a significant portion of mycovirus genes have unknown functions, some of them may serve as yet unidentified vector transmission-associated proteins, warranting further investigation.

Evolutionary connections between mycoviruses and plant and animal viruses hint at possible vector-mediated routes as well. It is undisputed that vector-borne transmission observed in plant and animal viruses has evolved to allow spatial transmission, as well as temporal one in the case of persistent transmission. Mycoviruses share families with plant and animal viruses which are transmitted by insects or other arthropods (Whitfield et al., 2015). For example, mycoreoviruses belong to the family of reoviruses and share similarities with plant reoviruses, which are persistently transmitted by insect vectors. In the case of Rosellinia necatrix mycoreovirus 3, it is closely related to animal coltiviruses, which are known to be transmitted by ticks in nature (Kanematsu et al., 2010). In addition, horizontal gene transfer between fungal and plant viruses might be more common than currently believed as discussed in a recent review paper (Dolja and Koonin, 2018; Ayllón and Vainio, 2023). It was found that capsid-less mycoviruses of *Hypoviridae* family have acquired genes from *Potyviridae* (Dolja and Koonin, 2018), which cover a large number of plant viruses that are vector-transmitted (Dombrovsky et al., 2012). Finally, two examples hint that fungal-feeding animals might act as a vector for mycoviral transmissions too. A mycovirus SsHADV1 was shown to not only transmit via fungivore (mycophagous) insect, *Lycoriella ingenua*, but its transmission to fungi was observed in a persistent way, where the mycovirus was able to replicate in the insect host and could be transmitted to offspring too via infected eggs (Liu et al., 2016). Similarly, *Thyreophagus corticalis*, a mite often observed to be feeding on chestnut cankers, was able to transmit CHV1 to another *C. parasitica* strain by fecal matter containing CHV1-infected mycelium (Simoni et al., 2014; Bouneb et al., 2016), offering an indirect non-persistent vector-mediated route of transmission. Finally, injuries by fungivores to the fungal mycelium might also promote viral transmission. Mechanical transmission via tissue injuries allows the release of virions, and this movement through mechanical forces promotes viral transmission in some soil-borne plant viruses (Petrov, 2016), suggesting that a similar mechanism might exist for mycoviruses. Together, these observations underscore the limited current knowledge on vector-mediated transmission of mycoviruses but lead us to hypothesize that it is indeed a viable possibility.

## 4 Plant-mediated transmission of mycoviruses: possibility in proximity

Various fungi colonize plant surface and endosphere establishing pathogenic, symbiotic, or commensal relationships with plants (Sapkota et al., 2015, 2017; Rojas et al., 2019). Plants and fungi that live in close association exchange diverse molecules and cellular contents (Bonfante and Genre, 2010). Many endophytic fungi inhabit plants, forming a range of interactions, from beneficial to asymptomatic. Plant root-inhabiting mycorrhizae is a widely studied example of such a close association and exchange of metabolites between two partners (Harrison, 1999). In contrast, plant pathogenic fungi invade plant tissues, interfere with plant growth, and in a few cases, secrete fungal toxins into the plant, resulting in economic losses in crop production. Sometimes, the same fungal species can change between pathogenic and non-pathogenic/endophytic depending on environmental factors or the host plant, a fact that has puzzled researchers (Wheeler et al., 2019). Interestingly, such transformation of fungal interaction with the host plant could, in a few cases, be linked to a mycovirus infection (Zhang et al., 2020; Zhou et al., 2021).

Recent virome studies suggest that plants and fungi host diverse viromes and are often infected by more than one type of virus at a time (Rumbou et al., 2021). When a fungus is in close association with a plant, the two viromes come in close contact within the same plant, and this could result in complex interactions. The two virus communities could complement each other to overcome cross-kingdom barriers leading to the replication and transmission of fungal viruses in plants as well as plant viruses in fungi. Several fungi form close relationships with plants and even exchange molecules as well as primary and secondary metabolites as a form of crosstalk, thus an exchange of viruses would be a natural next step. It has been shown that a few plant viruses are persistently transmitted by fungi (Campbell, 1996). This might also hold true in the opposite direction, where mycoviruses could potentially replicate in plants. In at least one report, it has been shown that a mycovirus from an endophytic fungus was able to replicate in plants (Nerva et al., 2017). Although such reports are limited, they suggest that few mycoviruses might cross the fungal-plant inter-kingdom boundary (Andika et al., 2017).

Many mycoviruses share families with plant viruses such as *Partivididae, Chrysoviridae, Endornaviridae.* In addition, several phylogenetic analyses of fungal and plant viruses often show close relationships confirming the recent divergence or cross-transmission of the viruses between fungi and plants (Lefeuvre et al., 2019). Most of these mycoviruses have adapted to life in fungal cells and lost the movement proteins used by plant viruses to achieve cell-to-cell movement through plasmodesmata in plants (Ayllón and Vainio, 2023). In contrast, movement of mycoviruses between fungal cells occurs naturally, as hyphae share a near-continuous cytoplasm, which is why movement proteins in mycoviruses are considered redundant for life within fungal cells. However, to establish systemic infection in plants, mycoviruses might be able to use the movement protein of other viruses either directly or indirectly. A recent example of such synergism between fungal and plant viruses has been reported (Bian et al., 2020), where a plant virus provided a movement protein function to a mycovirus, enabling its spread within a plant. Subsequently, it was demonstrated that another fungus could pick up the mycovirus, implying the existence of such a mechanism in nature. In addition, a few mycoviruses have retained the movement protein (Ayllón and Vainio, 2023). It was postulated that the mycoviruses encoding movement protein originated from a recent cross-kingdom virus transmission event from plants to fungi, where the movement protein became not functional (Ruiz-Padilla et al., 2021). However, the retention of the gene encoding movement protein in a few mycoviruses also suggests the possibility of simultaneous viral infection of both plants and fungi. Finally, reports of mycoviruses replicating in plants (Bian et al., 2020) indicate that the exchange of viruses might occur between fungi and plants via mutual interactions among the virome members, where one virus mediates the movement and transmission of another.

## 5 Conclusion and future directions

In this perspective, we have tried to shed light on the transmission of mycoviruses and to explore the possibility of vectors such as insects or other fungivores, as well as plants, as mediators of mycovirus transmission. Mycovirus transmission appears to be more widespread than what is expected from vertical and horizontal transmission via hyphal contact, restricted to vegetatively compatible strains, which might be due to unknown transmission pathways. If vector-borne mycoviral transmission occurs, it is expected to be crucial for understanding the diversity and ecology of mycoviruses, as it represents a mechanism evolved in other kingdoms to allow the transmission of viruses across spatial and temporal scales. Future studies on potential fungivore-mediated transmission of mycoviruses between fungi are necessary. Finally, several fungi living close to plants, or even within plants, might have implications for plant-mediated transmission of viruses, which could result from direct or indirect effects of plant virus synergistic interactions with mycoviruses. Collectively, while currently there is limited evidence of non-canonical transmission of mycoviruses, several exceptions are documented, and possibilities for new, unexplored routes are suggested. These potential transmission routes may be utilized infrequently, but with dramatic consequences, warranting further studies. In the context of mycoviruses used as biosolutions, the knowledge and understanding of mycovirus transmission routes are essential.

## Data availability statement

The original contributions presented in the study are included in the article/supplementary material, further inquiries can be directed to the corresponding author.

## Author contributions

ŽB: Writing – original draft, Writing – review & editing. AW: Writing – review & editing. RS: Conceptualization, Methodology, Visualization, Writing – original draft, Writing – review & editing.
